# Cerebrospinal fluid fatty acids are better predictors of mild cognitive impairment than Aβ_42_/tau ratio

**DOI:** 10.1002/alz70856_102952

**Published:** 2025-12-26

**Authors:** Jacob Apibilla Ayembilla, Yannick Joel Wadop Ngouongo, Biniyam A. Ayele, Alice Nono Djotsa, Muralidharan Sargurupremraj, Xueqiu Jian, Alfred Kongnyu NJAMNSHI, Sudha Seshadri, Jayandra Jung Himali, Bernard Fongang, Alfred N. Fonteh

**Affiliations:** ^1^ Accra Technical University, Accra, Greater Accra Region, Ghana; ^2^ Glenn Biggs Institute for Neurodegenerative Diseases, University of Texas Health Science Center at San Antonio, San Antonio, TX, USA; ^3^ Global Brain Health Institute, San Francisco, CA, USA; ^4^ John P. Hussman Institute for Human Genomics, University of Miami Miller School of Medicine, Miami, FL, USA, Miami, FL, USA; ^5^ Addis Ababa University, Addis Ababa, Addis Ababa, Ethiopia; ^6^ Section of Health Services Research, Baylor College of Medicine, Houston, TX, USA; ^7^ UT Health San Antonio, Bordeaux, TX, USA; ^8^ University of Texas Health Science Center at San Antonio, San Antonio, TX, USA; ^9^ University of Bordeaux, Bordeaux, France; ^10^ The University of Texas Health Science Center at San Antonio, San Antonio, TX, USA; ^11^ Brain Research Africa Initiative, Yaounde, Centre, Cameroon; ^12^ South Texas Alzheimer's Disease Research Center, San Antonio, TX, USA; ^13^ University of Texas Health San Antonio, San Antonio, TX, USA; ^14^ Glenn Biggs Institute for Alzheimer's & Neurodegenerative Diseases, San Antonio, TX, USA; ^15^ Department of Neurology, Boston University School of Medicine, Boston, MA, USA; ^16^ Graduate School of Biomedical Sciences, University of Texas Health Science Center, San Antonio, TX, USA; ^17^ Framingham Heart Study, NHLBI, Framingham, MA, USA; ^18^ University of Texas Health, School of Public Health, Houston, TX, USA; ^19^ Boston University, Boston, MA, USA; ^20^ Department of Neurology, University of Texas Health Sciences Center, San Antonio, TX, USA; ^21^ Glenn Biggs Institute for Alzheimer's and Neurodegenerative Diseases and Department of Population Health Sciences, San Antonio, TX, USA; ^22^ Boston University and the NHLBI's Framingham Heart Study, Boston, MA, USA; ^23^ The National Heart, Lung, and Blood Institute's Framingham Heart Study, Framingham, MA, USA; ^24^ Department of Neurology, UT Health San Antonio, 7703 Floyd Curl Drive, San Antonio, TX, USA; ^25^ Glenn Biggs Institute for Alzheimer's & Neurodegenerative Diseases, University of Texas Health Sciences Center at San Antonio, San Antonio, TX, USA; ^26^ Boston University School of Medicine, Boston, MA, USA; ^27^ Glenn Biggs Institute for Alzheimer's & Neurodegenerative Diseases, University of Texas Health Science Center, San Antonio, TX, USA; ^28^ Framingham Heart Study, Framingham, MA, USA; ^29^ The Framingham Heart Study, Framingham, MA, USA; ^30^ Department of Population Health Sciences, UT Health San Antonio, San Antonio, TX, USA; ^31^ Department of Population Health Sciences, University of Texas Health Sciences Center, San Antonio, TX, USA; ^32^ Glenn Biggs Institute for Alzheimer's & Neurodegenerative Diseases, University of Texas Health San Antonio, San Antonio, TX, USA; ^33^ Boston University School of Public Health, Boston, MA, USA; ^34^ Department of Neurology, Boston University Chobanian & Avedisian School of Medicine, Boston, MA, USA; ^35^ Glenn Biggs Institute for Alzheimer's & Neurodegenerative Diseases, The University of Texas Health Science Center at San Antonio, San Antonio, TX, USA; ^36^ Department of Population Health Sciences, The University of Texas Health Science Center at San Antonio, San Antonio, TX, USA; ^37^ Department of Biochemistry and Structural Biology, The University of Texas Health Science Center at San Antonio, San Antonio, TX, USA; ^38^ Huntington Medical Research Institutes, Pasadena, CA, USA; ^39^ Keck School of Medicine, University of Southern California, Los Angeles, CA, USA

## Abstract

**Background:**

Although fatty acids are integral structural components of neuronal membranes, knowledge of their metabolism and contribution to cognition is sparse and limited. This study aimed to evaluate the diagnostic performance of fatty acids in cerebrospinal fluid (CSF) fractions in the differential diagnosis of mild cognitive impairment (MCI) from cognitively unimpaired (CU) older adults.

**Method:**

Saturated fatty acids (SAFA), monounsaturated fatty acids (MUFA), and polyunsaturated fatty acids (PUFA) from three CSF fractions (unesterified, supernatant fluid and nanoparticles) were obtained from the Huntington Medical Research Institutes (HMRI) Aging Study. The data consisted of 68 CU and 38 MCI. The socio‐demographic data were summarized where continuous variables were reported as mean ± SD and categorical variables as counts and percentages. Multivariable binary logistic regression analysis was performed to differentiat CU from MCI. The best fitting CSF fatty acids biomarker was integrated with CSF Aβ_42_, tau, and Aβ_42_/tau ratio to assess their impact on the diagnostic performance. The model was adjusted for covariates; age, sex, smoking status, hypertension, diabetes, and APOE genotype. ROC curves were generated for the top 10 CSF fatty acids based on the area under the curve (AUC), sensitivity, and specificity and statistical significance differences between the groups using the DeLong test.

**Result:**

The palmitoleic acid/palmitic acid ratio, alpha‐linolenic acid, and palmitoleic acid in CSF unesterified, supernatant fluid, and nanoparticles fraction respectively effectively differentiated MCI from CU than Aβ, tau, and Aβ/tau with AUC of 0.70, 0.66 and 0.71 respectively which were significantly different from the AUC of Aβ_42_, tau and Aβ_42_/tau ratio (0.53, 0.54, 0.55) respectively. Moreover, integrating a panel of these fatty acids with Aβ_42_/tau significantly improved its diagnostic accuracy. Age, sex, smoking, hypertension, APOE genotype and diabetes did not influence the model performance.

**Conclusion:**

Our study suggests that changes in fatty acid metabolism precede abnormalities in amyloid and tau in MCI. Thus, strategies that regulate fatty acid metabolism may stem off early cognitive decline in an older population.

